# Influenzal Endocarditis

**Published:** 1906-01-06

**Authors:** 


### Hospital Clinics.

### INFLUENZAL ENDOCARDITIS.

It has long been known that the endocardium, especially the valvular endocardium, is very susceptible to microbic assault, and the tale of organisms accepted as occasional causes of malignant endocarditis is a long one. Dr. T. J. Horder [1] has recently published an account of two patients in whose blood the influenza bacillus was found several weeks before death; and since they are the first two cases of the kind which have been positively identified before death, it is worth while to reproduce briefly the clinical phenomena characterising the lesion. The first patient was a man of thirty-one years, a draper's assistant. Thirteen years before the onset of the fatal illness he had been attacked by influenza, followed by rheumatic fever, the whole illness lasting three months. After this illness he was told that he had a weak heart. Six years later he had to take to his bed for three weeks on account of feeling generally run down, and three years later again underwent a second illness, probably acute rheumatism, which lasted for ten weeks, and left him with an enlarged heart. From this date he had continued at work until ten weeks before his admission to hospital, when he began to ail again, complaining of weakness, sweating at night, and slight pain over the heart. For four weeks he had been unable to work, and had lost weight. These symptoms continued until the day of his admission, when he was suddenly seized with severe pain situated between the shoulders and extending to the neck and head. He was found to be a fairly nourished man, with a rapid pulse and rather hurried respirations. Physical examination revealed nothing abnormal in the lungs, but the heart gave the usual signs of aortic regurgitation, with hypertrophy of the left ventricle. There was no œdema nor any palpable enlargement of the spleen; the type of the fever was quotidian intermittent. The patient gradually lost flesh and colour during the month following his admission, at the end of which interval he suddenly developed a hemiplegia affecting the limbs, face, and tongue. This was accompanied by conjugate deviation of the head and eyes to the right, and by left hemi-anæsthesia. There was no loss of consciousness, but severe headache, sweating, and fever followed the attack. Three weeks after the first seizure the opposite—that is to say the right—side was involved in a hemiplegia, which ended fatally in two days. Post-mortem the right middle cerebral artery was found to be plugged by an embolus which had given rise to a considerable area of softening of the cortex and centrum ovale. The heart was very large, the left ventricle particularly being much hypertrophied. These appearances were undoubtedly due to a long-standing incompetence of the aortic valve, which was formed of two cusps only. The mitral cusps were thickened, but neither they nor the aortic cusps showed any signs of recent endocarditis. But on the wall of the left auricle, just above the base of the anterior mitral cusp, was a single mass of newly-formed tissue, slightly pedunculated, firmly attached to the endocardium, dark red in colour, smooth, and rounded in outline. This mass had penetrated the auricular wall, and had produced a patch of small granulations at the base of the anterior aortic cusp.

Blood-cultures were undertaken on four occasions during the life of this patient. On each occasion four cubic centimetres of blood were withdrawn from a vein at the bend of the elbow under the strictest aseptic precautions, and with this blood tubes of broth and of agar-agar were inoculated. In each case there resulted colonies of a small bacillus morphologically, and in its staining reactions, indistinguishable from the influenza bacillus, and, like it, not pathogenic to rabbits and guinea-pigs.

The second patient was a boy of thirteen, who had been a delicate child, though unaffected by any serious illness. Twelve months before his admission to hospital it was discovered (on the occasion of chloroform administration for the purpose of performing circumcision) that he had a "weak heart," but he had attended school regularly except for the last three weeks. His illness was not marked by any acute symptoms, but consisted of listlessness, abdominal pain, and a tendency to vomit after his meals. He was an anæmic, nervous boy, whose only objective signs of disease were a certain degree of fever, and evidence of mitral disease consisting in a loud, rough systolic murmur heard at the heart's apex and conducted into the axilla. The pulse was rather rapid; the spleen could not be felt, nor was there any albuminuria or dropsy. The vomiting occurred once or twice during the week following admission, but then ceased, and with it the abdominal pain came to an end. A month after admission the systolic murmur became musical for a week, and then reverted to its previous character. There was little alteration in the general condition, although the patient became somewhat thinner and more anæmic; but he was not very ill, for he was able to take part in the Christmas festivities of the ward. Twice more the musical character of the murmur came and went, discounting by its evanescent appearance the apparent improvement suggested by a quiescence of the fever which occurred at this time. In the third month of the illness the fever began to take a higher range, and there occurred a slight rigor, followed by vomiting. The anæmia became accentuated, and the patient generally more ill. A week later a second rigor was noted, and at about this time albuminuria made its first appearance; œdema of the legs set in, and the urine became scanty; the boy began to wander, grew rapidly weaker, and died three months after the onset of the illness. Post-mortem the heart was found to be enlarged; the mitral valve was stenosed, obviously by an endocarditis of distant date, while sprouting from the posterior aspect of the fused mitral cusps was a mass of rounded "vegetation."

This mass obstructed the orifice in such a way as to suggest that it was the cause of the musical systolic murmur which had been a feature of the case. There were old infarctions in the spleen and kidneys. In this case, as in the other, cultivation of the blood during life revealed the presence in the circulation of the influenza bacillus; and microscopic examination of the vegetation confirmed the case as one of malignant influenzal endocarditis.

In his conclusion Dr. Horder observes that the condition present in these two cases was an influenzal septicæmia, the cases standing in striking contrast with the ordinary run of influenzas, in the course of which the bacillus does not enter the circulation. The illness in each case began insidiously, and without any symptoms suggestive of the ordinary influenzal attack; the course of the disease was protracted, and in each case the influenzal infection was grafted upon an endocardium damaged by previous attacks of rheumatism. The clinical features of the disease differ in no essential respects from those associated with the majority of instances of malignant endocarditis. Another point of interest is that whereas influenza as a general rule causes no leucocytosis, both the patients here considered showed a well-marked increase in the number of white cells. Touching the possibilities of treatment the author observes that at present it would appear as though the patient were doomed from the moment the diagnosis of influenzal endocarditis can be positively made. The influenza bacillus is a parasite so strictly human that no certain pathogenic effects have as yet been obtained by animal inoculation, and in consequence no actively immune serum can at present be expected. Dr. Horder states that if faced with another case of this kind he intends to try direct inoculation of the patient with dead cultures of the influenza bacillus, in the hope that by such a process of vaccination the infection may be combated by an increased resistance on the part of the patient.

[1] Paper read before Med. Chir. Soc., November 14, 1905.

